# Potential benefits of JAK inhibitor therapy in Blau syndrome: a case report

**DOI:** 10.3389/fimmu.2025.1665949

**Published:** 2026-01-12

**Authors:** Yu Wang, Xiao-juan Yu, Zhuoli Zhang

**Affiliations:** 1 Department of Rheumatology and Clinical Immunology, Peking University First Hospital, Beijing, China; 2 Department of Nephrology, Peking University First Hospital, Beijing, China

**Keywords:** Blau syndrome, JAK inhibitor, nephrology, pathology, rare

## Abstract

Blau syndrome is a rare autoinflammatory disorder caused by gain-of-function mutations in the NOD2 (nucleotide binding oligomerization domain containing 2 receptor) gene. Blau Syndrome presents with the diagnostic triad of chronic polyarticular synovitis, recurrent uveitis, and dermatitis. Notably, patients often develop systemic granulomatous inflammation affecting multiple organs, particularly the kidney and liver. Here we report a case of Blau syndrome presented with early-onset arthritis, uveitis, and renal involvement, evidenced by granulomas tubulointerstitial nephritis. Genetic testing showed a pathogenic p.R334W NOD2 mutation demonstrating constitutive NF-κB activation and excessive proinflammatory cytokine production. While initial corticosteroid therapy improved articular and ocular symptoms, renal dysfunction persisted until baricitinib (4 mg/day) initiation, which rapidly normalized renal function and permitted steroid tapering. Granulomatous inflammation in Blau syndrome is mediated by IFN-γ and sustained JAK-STAT activation, making JAK1/2 inhibition a rational therapeutic target. Although TNF-α inhibitors show efficacy in some cases, our experience supports baricitinib’s potential for refractory disease, particularly with renal involvement. Baricitinib can offer distinct advantages over biologics and effectively downregulates inflammation in Blau syndrome.

## Introduction

Systemic auto-inflammatory diseases (SAIDs) are a growing group of disorders caused by a dysregulation of the innate immune system. Furthermore, it has been observed that 40-60% of patients exhibiting symptoms consistent with the typical presentations of SAIDs do not receive a definitive diagnosis. Although corticosteroids is the most used drug due to anti-inflammatory effect, its use seems insufficient in Blau syndrome. 68% of the patients with ocular manifestations needed a combination therapy with immunosuppressive agents and/or biological drugs (1). Methotrexate, thalidomide, sulfasalazine, mycophenolate et al. were used to treat Blau syndrome, however, the efficacy is difficult to ascertain and may have side effects. Considering Blau syndrome as a poor factor for uveitis, monoclonal antibody drug such as adalimumab and infliximab is preferred biological therapies, and short-term systemic corticosteroid should be considered only as bridging therapy. Here, we present a case of successful treatment of baricitinib in combination with steroids in a patient diagnosed with early onset sarcoidosis (EOS) or Blau syndrome with a NOD2 gene mutation.

## Case report

A 22-year-old male presented with a 15-year history of progressive arthritis and joint deformities, accompanied by recent-onset blurred vision and elevated serum creatine persisting for 3 months. Previous treatment with traditional Chinese herbs had proven ineffective.

**Figure 1:**
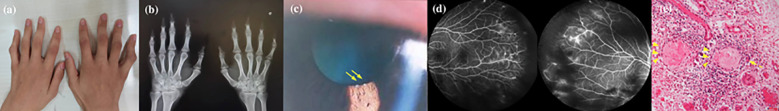
**(a)** Symmetric flexion contractures of bilateral proximal interphalangeal joints 2 to 5. **(b)** Joint space narrowing and sclerosis on X-ray. **(c)** off-white deposits-mutton fat keratic precipitates on the inner corneal surface in the right eye (arrows). **(d)** Bilateral retinal vasculitis on fluorescein angiography. **(e)** Pathology showed granulomatous tubulointerstitial nephritis. Multiple focal and patchy infiltrate of lymphocytes, monocytes, and some plasma cells in the renal interstitium with fibrosis, and granulomatous structures (arrowhead), accompanied by multinucleated giant cell reactions (dotted arrow).

Physical examination revealed multiple joint deformities, particularly in the hands, with no associated skin changes. Radiographs demonstrated joint space narrowing, without erosion (**Figure 1A, B**). Ophthalmological evaluation identified the presence of bilateral granulomatous anterior uveitis (Panel c and d), evidenced by mutton-fat keratic precipitates, a positive Tyndall phenomenon and visual acuity of 0.6 and 0.8 in the right and left eyes, respectively. Intraocular pressure was normal. Urinalysis showed proteinuria with microalbumin 87.3 mg/L (0–19) and microscopic hematuria (10 red blood cells/HP). Serum creatinine (172 μmol/L) and urea (8.77 mmol/L) were elevated, alongside increased erythrocyte sedimentation rate (ESR) of 44 mm/h and C-reactive protein (CRP) of 11.2 mg/L. Autoantibody screening was negative. The serum angiotensin-converting enzyme was found to be within normal parameters. Infectious etiologies (cytomegalovirus, HIV and syphilis) and tuberculosis (interferon-gamma release assay and PPD) were excluded. A subsequent renal biopsy revealed non-necrotizing granuloma (Panel E).

**Figure 2:**
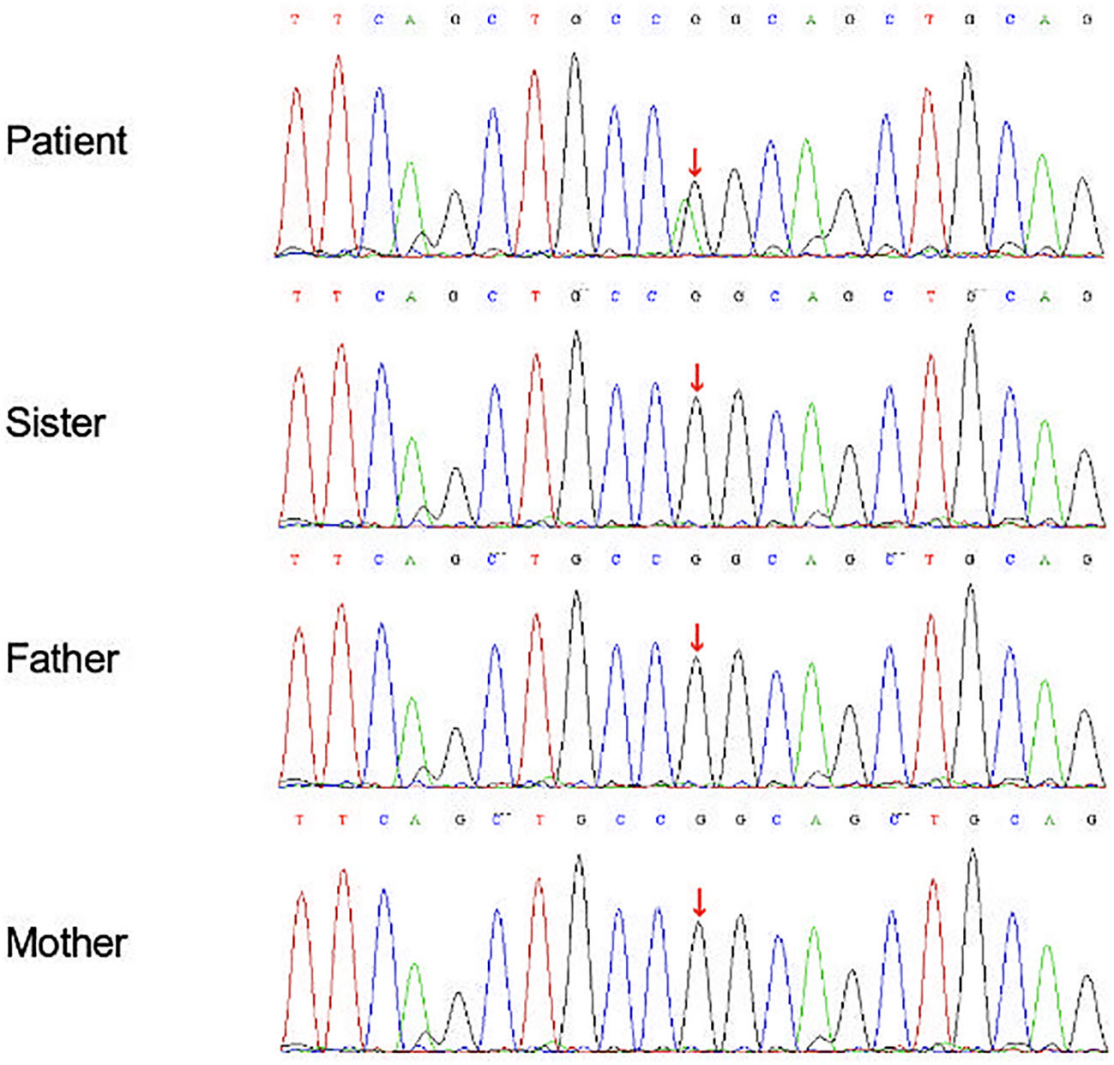
Gene sequencing of the NOD2 gene by sanger whole genome sequencing (WGS) for the patient and his family. The patient had a *de-novo* heterozygous mutation in the NOD2 gene (c.1001G>A, chr16:50744823 p. Arg334Gln). The gene analysis of his parents and sister were normal.

Given the triad of arthritis, uveitis, and systemic granulomatous involvement, Blau syndrome and sarcoidosis were considered. Subsequent gene screening revealed a *de novo* NOD2 mutation (c.1001G>A, chr16:50744823 p. Arg334Gln), absent in his parents and sister, confirming a diagnosis of EOS with NOD2 mutation (**Figure 2**).

**Figure 3:**
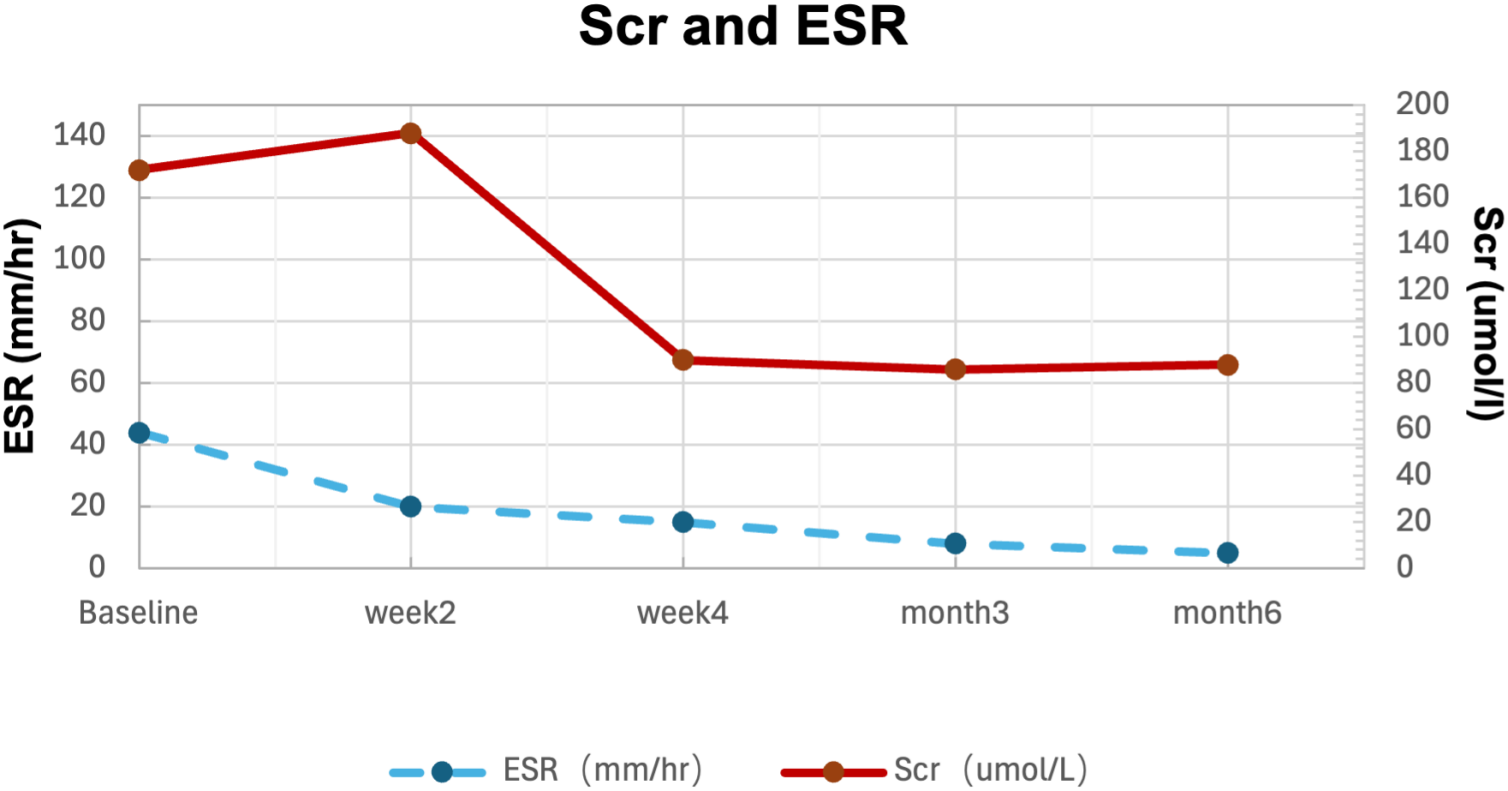
Serum creatine and ESR changes with timing in response to treatments.

Initial therapy with prednisone 1mg/kg/d improved uveitis and arthritis within 2 weeks, but Scr rose to 188 umol/L. Since he refused injectable therapies, baricitinib 4mg/day was initiated as a steroid-sparing agent based on its JAK1/2 inhibitory mechanism. Within two weeks, arthritis and uveitis resolved completely, serum Scr normalized (90 umol/L), and inflammatory markers (ESR <15 mm/hour, and CRP <5mg/L) remained suppressed. Over six months, prednisone was successfully tapered to 5mg daily without disease relapse or any side effects (**Figure 3**; **Table 1**).

**Table 1:** Laboratory results and treatment throughout follow-up.

	Baseline	week2	week4	month3	month6	Normal range
ESR (mm/hour)	44	20	15	8	5	0-20
CRP (mg/L)	11.2	8	6	4	5	<10
WBC (/ul)	8240	9800	6900	4230	4780	4000-10000
Neutrophils (/ul)	7200	8230	4820	4130	3720	1500-8000
Lymphocytes (/ul)	950	900	850	740	780	700-3100
Hemoglobin (g/l)	110	115	114	120	118	130-175
Platelets (/ul)	280,000	275,000	208,000	214,000	202,000	100,000-300,000
Scr (umol/L)	172	188	90	86	88	65.4-119.3
urinary microalbumin (mg/L)	87.3	65.2	19	17	15	0-19
urinary red blood cells (/HP)	10	5	2	3	2	0-3
prednisone (mg/d)	60	55	50	30	5	
Baricitinib (mg/d)	0	4	4	4	4	

## Discussion

Blau syndrome (2) is a rare autosomal dominant disorder characterized by clinical triad of arthritis, uveitis, skin rash and granulomatous inflammation. The causative gene CARD15/NOD2 was identified in 2001, and encodes a protein involved in innate immune regulation, with shared pathogenic mechanisms in inflammatory bowel disease (3). The NOD2 gene encodes three functional domains: 2 caspase recruitment domains (CARDs), a central nucleotide oligomerization domain (NOD/NACHT) and a C-terminal leucine-rich repeat region (LRR).

In the present case, genetic analysis revealed a pathogenic NOD2 gain-of-function mutation (p.R334W), which has been previously associated with panuveitis (4). This mutation induces constitutive activation of NF-κB pathway, independent of peptidoglycan-independent mechanism, resulting in excessive production of proinflammatory cytokines (5). Furthermore, IFN-γ has been identified as a critical mediator in the pathogenesis of granulomatous inflammation, a hallmark feature of this disorder. Blau syndrome typically manifests in early childhood, with classical triad of polyarticular synovitis, granulomatous uveitis and papular rash. Systemic involvement may also extend to multiple organs, including renal (glomerulonephritis), hepatic (granulomatous hepatitis), neurological (cranial neuropathies), and vascular (arteritis) systems. Ocular manifestations often progress relentlessly, potentially leading to severe visual impairment. The same phenotype occurring without family history is termed Early Onset Sarcoidosis, though both inherited and sporadic forms are now commonly classified under the umbrella term Blau syndrome. The differential diagnosis of Blau syndrome should include juvenile idiopathic arthritis, especially in cases presenting with atypical features such as camptodactyly, cutaneous eruptions, or granulomatous panuveitis, particularly with a positive family history of similar symptoms. Early diagnosis is crucial to prevent irreversible end-organ damage, particularly vision loss from progressive ocular inflammation.

Pediatric anterior uveitis may frequently present asymptomatically in until advanced, irreversible sight-threatening complications develop. While anterior chamber inflammation is often difficult to be detected, the presence of mutton-fat keratic precipitates can be seen with the naked eyes. The presence of these large deposit (macroscopic aggregates of polymorphonuclear cells, lymphocytes, and epithelioid cells) strongly suggests a pathognomonic feature of granulomatous disease and warrants immediate intervention (2). Current therapeutic strategies include corticosteroids, interlukin-1 inhibitors, and TNF-α antagonists, with varying degrees of efficacy reported in a few clinical studies (6). Emerging evidence suggests the JAK inhibitors may represent a promising therapeutic alternative for refractory cases. Tofacitinib has shown clinical efficacy in previous reports (7), with one documented case of successful maintenance therapy following discontinuation (8).

In our patient, while corticosteroids therapy achieved partial improvement in arthritis and uveitis within the first week, renal dysfunction persisted. Initiation of baricitinib 4mg/day resulted in rapid resolution of both articular and ocular manifestations, with concomitant normalization of renal function. Sustained clinical remission was achieved, enabling prednisone tapering to 5mg/day by 6 months of follow-up.

Blau syndrome represents a severe autoinflammatory disorder characterized by frequent treatment resistance. The absence of established therapeutic guidelines, compounded by the rarity of the disease, necessitates reliance on expert consensus for clinical management. Although corticosteroids remain the most frequently employed first-line therapy, they often prove inadequate for complete disease control, particularly in cases with multiorgan involvement (1). Notably, ocular manifestations affect 32% of patients with Blau syndrome frequently result in permanent visual impairment. Patients with systemic involvement typically require aggressive immunosuppressive regimens, with biologic agents reserved for refractory cases. While evidence remains limited for IL-1, IL-6, and JAK inhibitors, TNF-α antagonists have demonstrated efficacy in inducing hepatic remission, in 5 Blau syndrome patients as reported by Sinharay et al (9). Our findings align with previous reports of Baricitinib efficacy in a treatment-resistant Blau syndrome under after methotrexate, adalimumab, and tofacitinib failure (8), particularly in cases with renal granulomatous involvement. As a selective inhibition of JAK1/JAK2 inhibitor, the mechanism of baricitinib can directly targets the pathological activation of granulomatous inflammation, in modulating the dysregulated inflammatory cascade in Blau syndrome, offering a targeted therapeutic approach for this challenging condition.

## Conclusion

Baricitinib emerges as a viable therapeutic option for refractory Blau syndrome, particularly in cases with renal granulomatous involvement. Its demonstrated efficacy in achieving rapid clinical remission and steroid-sparing effects warrants further investigation through controlled clinical studies to establish optimal dosing protocols and long-term safety profiles in this rare disease population.

Serum creatine (solid line) and ESR (dotted line) levels. Left arrow indicates prednisone 60mg/d; right arrow indicates Baricitinib 4mg/d.

## Data Availability

The raw data supporting the conclusions of this article will be made available by the authors, without undue reservation.
